# Assessment of the global healthcare industry during COVID-19 pandemic: A content analysis approach

**DOI:** 10.12688/f1000research.132486.2

**Published:** 2024-04-24

**Authors:** Malik Ladki, Latifa Daher, Robert Abou Chacra, Elie Kassis, Chady Ayrout, Hadi Moubayed, Amal Abbas, Nahed Boudani, Ralph A Doumit, Sylvana Bitar, Annie Kizirian, Rola Hasna, Darine Barakat, Wissam H. Faour

**Affiliations:** 1 The University of Texas Medical Branch, Galveston, Texas, USA; 2 EMBA Program, Adnan Kassar School of Business, Lebanese American University, Beirut, Lebanon; 3 Gilbert and Rose-Marie Chagoury School of Medicine, Lebanese American University, Byblos, Lebanon

**Keywords:** content analysis, periodicals, healthcare, COVID-19

## Abstract

**Background:**

Content analysis (CA) is an investigative research tool used in healthcare studies to identify imminent challenges while providing lead time for preparedness measures. The objective of this study is to report on the challenges confronting the global healthcare industry.

**Methods:**

This study used the conventional CA “bottom-up” approach to a quantitative data. CA is a research technique that uses the qualitative research method and word counts to evaluate communication outcomes. A group of executive master’s in business administration (EMBA) and medical students championed the study. Using 13 periodicals as the data sources, researchers conducted online keyword searches for all articles, titles, and abstracts containing the word “healthcare”. The collected data was gathered from five international, four Middle Eastern, and four Lebanese periodicals from January 1, 2021 to December 31, 2021.

**Results:**

CA results indicate remarkable domination of keywords “COVID-19 health impact” as a major worldwide theme. Significant differences were noticeable among subcategories but remained related to COVID-19 in particular, social responsibilities, and research and development. Topics such as comorbid illnesses, social responsibility, healthcare logistics, and the well-being of healthcare service providers were among the least covered topics.

**Conclusion:**

Our findings emphasize the importance of global media and their coverage of healthcare issues on the types of healthcare topics ( *e.g.*, vaccines, drugs…) covered in the Lebanese periodicals in our study. Thus, global healthcare media are the main source for Lebanese periodicals reporting healthcare issues rather than the Lebanese healthcare authorities. Therefore, there is need for the development of a more efficient technology-driven healthcare management system. At the global level, there is a need to develop a step-by-step action-oriented strategic initiative to respond to emerging pandemics.

**Abbreviations**
COVID *-*19: Coronavirus disease 2019
CA: content analysis
R&D: Research and development

## Introduction

Content analysis (CA) is a multipurpose research technique that deals with the objective, systematic, quantitative, qualitative, and general description of the manifest and latent content of communication. ^1^^,^^2^ As a research tool, CA determines the reoccurrence of words, themes, or concepts in qualitative data. ^3^ It is a research technique that uses a set of formalized procedures to classify communication to draw valid inferences. CA provides researchers with the opportunity to unobtrusively study the values, intentions, and ideologies of any group. ^4^^–^^8^ Thus, CA enables researchers to quantify and analyze the presence, meanings, and relationships of concepts, words, or themes in various communication mediums.

Regardless of the diversity of CA definitions, many researchers agree on the objectivity, systematic, and generality of any CA analysis. ^9^ Objectivity is maintained when data is collected based on explicitly formulated rules. Researchers tend to use professional judgment in making decisions about their data. Thus, enabling researcher biases or subjectivity to creep into the investigation can skew the results. It is important to minimize biases by establishing and following strict procedures and thus enabling those who duplicate the study to achieve similar results. The systematic requirement is the inclusion and exclusion of content according to a set of applied rules where the investigator biases are eliminated and multiple coders follow established procedures to ensure outcome validity and reliability. Generality entails that the findings must have theoretical relevance and is achieved when some form of harmony or similarity is found when findings are compared.

Most CA investigations center around three distinct approaches: conventional, directed, or summative approaches. ^4^ Regardless of the approach, the outcome of CA is to interpret communication intents or meanings. Coding schemes represent the difference among the approaches, and the conventional technique is the most common coding technique where categories originate from words. Whereas in the directed approach there is a hypothesis and a theory to guide researchers to identify codes. In the summative approach, researchers count and compare words and then interpret the underlying theme.

The use of CA in health care literature is becoming prevalent. ^10^^–^^14^ A surge in the use of qualitative research is being witnessed in many fields including healthcare, medical, and biomedical literature. ^15^^,^^16^ Qualitative CA in healthcare research has been applied to draw interpretation from a variety of sources. ^17^^–^^20^ The application of CA in healthcare encompasses all disciplines including policies, ^21^ genomic analysis, ^22^ population health, ^23^ biomedical technology, ^24^ medical decision making. ^25^

Accordingly, content analysis was used to identify challenges in patients suffering of Hemophilia. ^26^ Also, it is also used to evaluate emergency and critical care in nursing settings. ^27^ Importantly, it is commonly used to evaluate the access to appropriate healthcare services and coverage. ^28^^,^^29^ Recently, content analysis evaluating stressors among health care found that 70% are experiencing burnout, which can hinder patient care. Finally, CA was used to evaluate professionalism among medical students and found that two core thematic aspects of professionalism on patients’ respect and physicians’ accountability. These findings highlight the important of CA in all aspects of the healthcare system.

The healthcare system throughout various regions confronted major challenges due to coronavirus disease 2019 (COVID-19) pandemic. The purpose of this study is to report on the challenges confronting the global healthcare industry. The study intends to enable health organizations, researchers, professionals, and governments to raise public awareness on the most challenging or critical healthcare issues confronting the industry throughout the various regions of the world.

## Methods

The international periodicals were selected among USA based periodicals since the authors concluded that USA media have strong influences worldwide. The listed US periodicals were also chosen since online access to the periodical archives are easily accessible through Lebanese American University (LAU) online library, as well as librarian support about the search tools through these periodicals. The regional periodicals were chosen from two countries that have major influence in the Arabic region: Saudi Arabia and Egypt. As Jordan is a nearby Arabic country to Lebanon and share similar economic situation, thus Jordan times was also preferred and selected. The Lebanese periodicals were chosen to cover the language diversity (Arabic, French and English) that exists in Lebanon and taking into consideration free online access to the periodical archives. The availability of free online access to the periodical archives was also a crucial criterion. In order to minimize bias, students or “coders” were trained in advance on data collection and how to search for data as part of their EMBA course on content analysis. Furthermore, each student was not allowed to interact with the other members in order to prevent judgment bias about the coding process and the collected data. Each student collected data individually and didn’t communicate with any of the students before submitting the data to the corresponding author. Data gathering and analysis was carried out using Excel 2016, and chart lines were made in order to assess the trends of categories among each periodicals on a monthly basis.

The investigators have gathered data from 13 of the periodicals in various parts of the world from January 1, 2021 to December 31, 2021. This study uses the conventional CA “bottom-up” approach to a qualitative data set. ^30^ This approach enables researchers to collect, sort, and categorize data to identify emerging patterns or to develop theories. ^31^ Briefly, in bottom-approach all the team members involved in data collection will work proactively in each step of the project execution process until the final goal of the project is reached. Therefore, all the team members are involved in decision-making about the process and the strategy to follow as well as the outcomes of the analysis.

The study was conducted by a collaborative research group of Executive Master in Business Administration (EMBA) and medical students at two universities. Students at the Lebanese American University (Beirut, Lebanon) and the University of Texas Medical Branch (Galveston, Texas) collaborated on this research initiative. The students were divided into three groups: The first group collected data from the following international periodicals: The New York Times, Financial Times, Boston Herald, The Wall Street Journal, and Los Angeles Times. The second group collected data from four of the Middle East’s top English printed periodicals: Saudi Gazette, The Jordan Times, Gulf Daily News, and Daily News Egypt. The third group collected data from four online Lebanese, two Arabic (Al Akhbar, An Nahar), one English (Daily Star) and one French (L’Orient Le Jour) periodicals ( Table 1). The underlying data is available through the online repository. ^42^

**Table 1.** Periodical names and investigation day.

U.S and British Periodicals		Middle East Periodicals		Lebanon Periodicals	
Name	Day	Name	Day	Name	Day
New York Times	Monday	Gulf Daily News	Thursday	Al Akhbar	Monday
Financial Times	Tuesday	Saudi Gazette	Friday	Al Nahar	Tuesday
Boston Herald	Wednesday	The Jordan Times	Saturday	Daily Star	Wednesday
Wall Street Journal	Thursday	Daily News Egypt	Sunday	L’Orient Le Jour	Thursday
LA Times	Friday				

### Training the coders and search procedure

Two weeks prior to the initiation of the coding process, coders went through several intensive training sessions. Over time and with ongoing practice, coders developed the technical skills of matching. The 13 coders were broken down into 3 clusters, cluster 1 (International periodicals) was composed of 5 coders, cluster 2 (ME) and cluster 3 (Lebanese), both clusters were composed of 4 coders each respectively. Coders visited the library where all periodicals for five years period was awaiting for them. Each coder was issued a specific periodical for one year. The coders were first instructed to select all healthcare related articles, then look for keywords related to “healthcare”. Once the keyword was identified it will be counted as one data point. As example, any article in the periodical containing a healthcare related keyword “Vaccine” it is consider as one data point for the vaccine keyword. Similarly keywords such as “Hospital” if it exists in the article it adds one point to the hospital keyword count. The presence of the word is counted once only. To improve data validity and reliability the number of times the keyword is repeated in the article was not counted. Data was collected from all periodicals once a week throughout the investigation period of 2021. Coders were trained, then assigned to investigate specific periodicals in each cluster. Regular meetings were held to monitor the coders’ compliance with the objectives of the study and the use of agreed upon coding schemes. Results represent data for each region of the world. Further, all regions were compressed together, and the global data set emerged. The result section of the compressed global data obtained after regrouping the keywords in sets resulted in 10 categories which represent the most frequently healthcare issues reposted in 2021.

### Sample

Using 13 periodicals as the data source, researchers conducted online keyword searches for all articles, titles, and abstracts containing the word “healthcare”. Researchers manually browsed through each periodical for the period of the study (January 1, 2021 to December 31, 2021) to identify all healthcare related articles. This procedure enabled researchers to compile a comprehensive data set of published communication containing the word “healthcare”. To eliminate the redundancy of collected data, the researchers systematically assigned a specific day as a source of input for each periodical ( Table 1).

### Procedure

Researchers have systematically analyzed the content of 13 periodicals once a week for a year. The data set produced the findings of the content from 676 days from within the year 2021. Researchers examined the context in which the occurrences of words, and phrases, related to “healthcare” and recorded then analyzed findings. “Healthcare” was used as major output for all countries due to COVID19 pandemic.

To ensure the validity and reliability of collected data, all coders were trained and then instructed to follow the following protocol:

(i) Establishing the document inclusion criteria (identifying documents based on the titles & tags of the articles, selecting the ones with topics related to healthcare).
(ii) Gathering documents through online scanning of the periodicals.
(iii) Identifying keywords by manually examining the content of the selected documents.
(iv) Developing coding criteria to ensure validity (being consistent in following coding rules, Table 2).
(v) Eliminating keywords with low-frequency counts (removing irrelevant keywords that are not fully related to healthcare ( *e.g.* salaries, climate changes).
(vi) Classifying the keywords into categories (introducing keywords into a particular category is based on their relevance to that category).
(vii) Drawing conclusions and generalizations (data analysis).

**Table 2.** Example: Training the coders on formation of categories (detailed description of the terms in each category are available in the repository).

Categories	Definition
**COVID health impact**	All words related to COVID impact like cases, deaths, infected, infections, virus, coronavirus … N=10,656.
**COVID social, economic & financial Impact**	All words related to the impact of COVID on the social, economic, financial sectors including school, tourism, travelers, companies, work … N=2627.
**COVID affected parties**	This includes all words related to patients, country, nation, excluding the healthcare staff … N=6621.
**Strategic action plan for COVID**	This includes all the preventive measures taken to prevent the spread of COVID in the Middle East … N=10,400.
**Healthcare human resources**	This includes all the staff of the hospitals, insurance, administration, nurses, drs, … N=2096.
**Social responsibility**	This is defined as any social & financial aids related words given to any country including donation, support … N=760.
**Healthcare logistics**	This includes words related to hospitals & healthcare operations & logistics such as ambulance, shipment, beds, equipment … N=1897.
**Healthcare future and R&D**	This relates to the pharmaceutical industries, biotechnology, research & medicines such as pharma companies, vaccine, doses, booster, Pfizer, … N=2599.
**Healthcare diseases**	This relates to words that reflect non COVID related diseases including Diabetes, autism, diet, obesity, … N=272.
**Healthcare system authority**	This includes the COVID emergency task force setting rules& regulations, preventive measures & guidelines words related to authorities, government, police, committee, members, president, … N=3560.

### Coding scheme

To come up with an understanding of the investigated research questions, the investigators recorded and analyzed the occurrences of words, phrases, and sentences related to “healthcare”. Based on data interactions, numerous categories were identified and then integrated into a framework comprising a trend line to answer the research questions ^32^ through an in-depth analysis of the most popular periodicals throughout the various regions of the world. By examining word frequency counts and categorizing data, our objectives were to provide a meaningful source of informational input for health organizations, researchers, professionals, and governments, and to raise public awareness on most challenging or critical healthcare issues.

### Coding

Selected periodicals represent some of the most widely circulated periodical in certain region of the world ( Table 2). All periodicals were available through on-line subscriptions at the libraries of participating universities. Data was collected on Excel 2016 and clustered into three groups (International periodicals, Middle Eastern periodicals, and Lebanese periodicals). The proportions of the keywords frequencies were then calculated and used to create the trend lines shown in the figures. Though the 13 periodicals were printed in three different languages (English, French, and Arabic), coders were versatile and fluent in the above foreign languages and were able to read, write, and analyze data.

Data was collected from all periodicals once a week throughout the investigation period of 2021. Coders were trained, then assigned to investigate specific periodicals in each cluster. Regular meetings were held to monitor the coders’ compliance with the objectives of the study and the use of agreed upon coding schemes. Results represent data for each region of the world. Further, all regions were compressed together, and the global data set emerged. The result section of the compressed global data obtained after regrouping the keywords in sets resulted in 10 categories which represent the most frequently reported healthcare issues during 2021 and are defined in Table 2.

## Results

### COVID-19 related impact occupied most of the news in international periodicals

In keeping with the effect of COVID-19 internationally, our data showed that international periodicals shared similar coverage about the improvements in COVID-19 in health care but further focused on the strategic plan against COVID-19 pandemic in addition to its social and economic impacts. For instance, the category “strategic action plan for COVID” showed the highest frequency percentage of 25.7%, followed by the category “COVID Health impact” with 21.3%, while the percentage of the “Health care future and research and development (R&D)” category was 14.2%. Furthermore, the categories covering social responsibility, healthcare logistics, and the well-being of healthcare service providers in addition to the social, economic, and financial impact of COVID-9 were also covered in all periodicals but at a lower frequency.

### COVID-19 health-related impact occupied most of the Middle East newspapers

Figure 2 shows the impact of COVID-19 on health systems, how it is a major concern and ranked first among all other topics in all chosen Middle Eastern journals including Gulf Daily News, Saudi Gazette, and The Jordan times, except for Daily News Egypt. Accordingly, the category “COVID health impact” showed the highest frequency percentage of 25.7%, followed by the category “Strategic plan for COVID” with a frequency percentage of 25.1%, and the category “COVID affected party” ranked third with a frequency percentage of 16.0%. While Daily News Egypt showed a different pattern where the strategic plan to fight COVID-19 was ranked first, COVID-19 health impact was still considered a topic of high importance and ranked third among other topics. All other topics including social responsibility, healthcare logistics, and the well-being of healthcare service providers in addition to the social, economic, and financial impact during COVID-19 were covered at a lower frequency with the highest being for the category “Healthcare system authority” with 8.6% and the category “Healthcare disease” showed the least frequency percentage of 0.7% in all these periodicals.

### COVID-19 health-related impact occupied most of the news in the national (Lebanese) periodicals

Figure 3 shows that the health impact of COVID-19 was a major concern and ranked first among the topic in the national periodicals including L’Orient Le jour, Al-Akhbar, and Daily Star throughout the year 2021. Furthermore, the finding corroborated and was similar to the data found in the international periodicals where the impact on health and strategic action plan for COVID-19 were among the most covered topic in these periodicals. Accordingly, the category “COVID health impact” showed the highest frequency percentage of 33%, followed by the category “Health care future an R&D” with a frequency percentage of 23%. Finally, the category “Strategic plan for COVID” was ranked third with a frequency percentage of 13%. Finally, all other topics including social responsibility, healthcare logistics, and the well-being of healthcare service providers in addition to social, economic, and financial impact of COVID-19 were not sufficiently covered in all these periodicals.

## Discussion

Healthcare systems worldwide faced major challenges during COVID-19 pandemic. At the beginning of the pandemic, healthcare systems worldwide were chaotically responding to the outbreak and its consequences. ^33^ While healthcare is mostly focused on providing proper treatment to infected patients, soon after, healthcare managers recognized that existing intervention policies are not serving their purpose. ^34^

Our data showed that international periodicals served as a source of COVID-19 data input for local and regional COVID-19 news. Our data concluded that international periodicals act as “trendsetters” whereas regional and local news were followers. Such finding is highlighted by the fact that coverage of “vaccines” and other keywords that mentioned in local and regional periodicals at the early stages of COVID-19 only started to appear after their appearance/coverage in the international media. Accordingly, we believe that low-income countries will continue to rely on developed countries to prioritize healthcare spending. This was expected by the authors given the scarcity of resources allocated to R&D COVID-19, in addition to the lack of policies and health task forces to face challenges such as COVID-19 pandemic.

**Figure 1.**
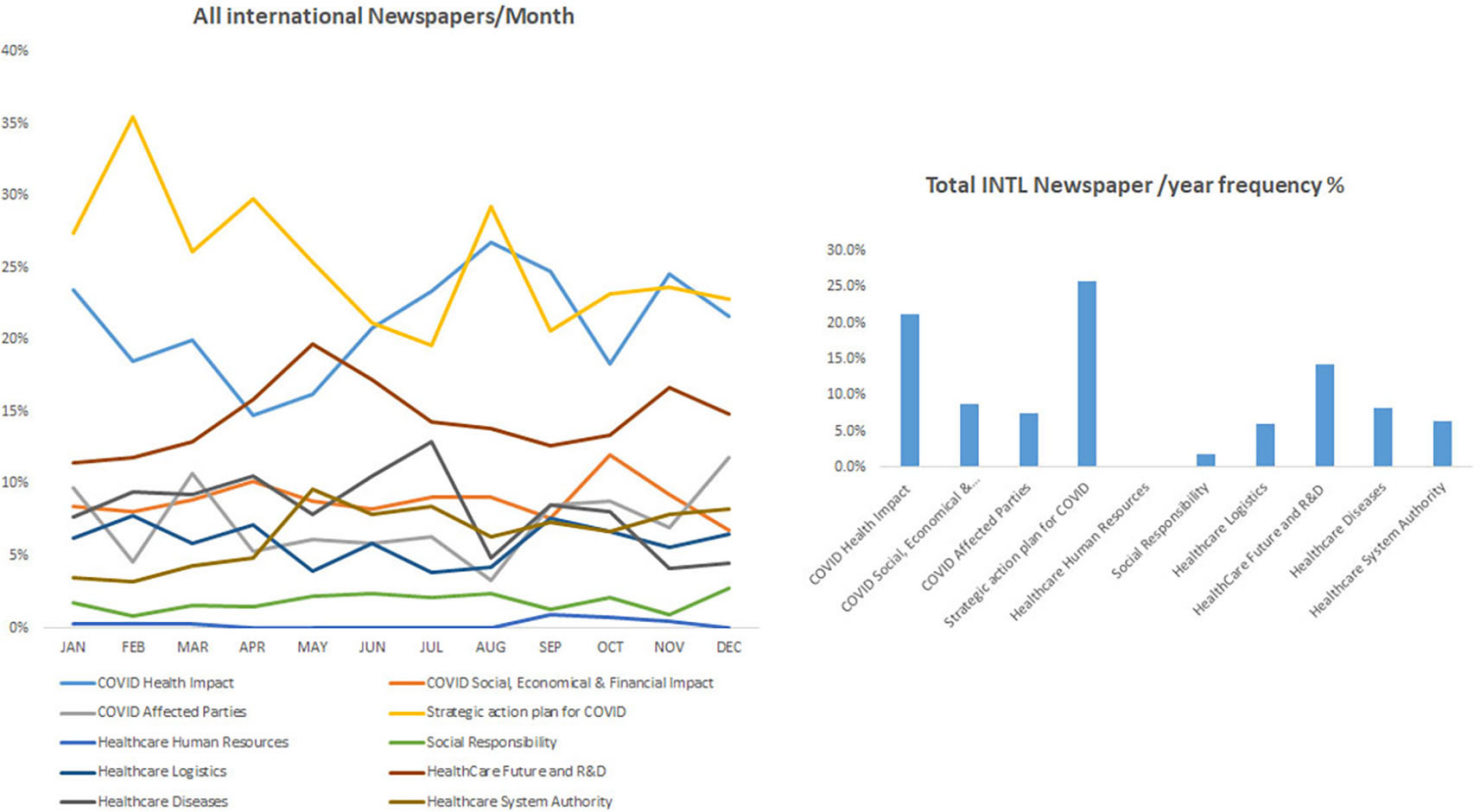
Trendline and frequency of categories of all international newspapers per month and year respectively.

**Figure 2.**
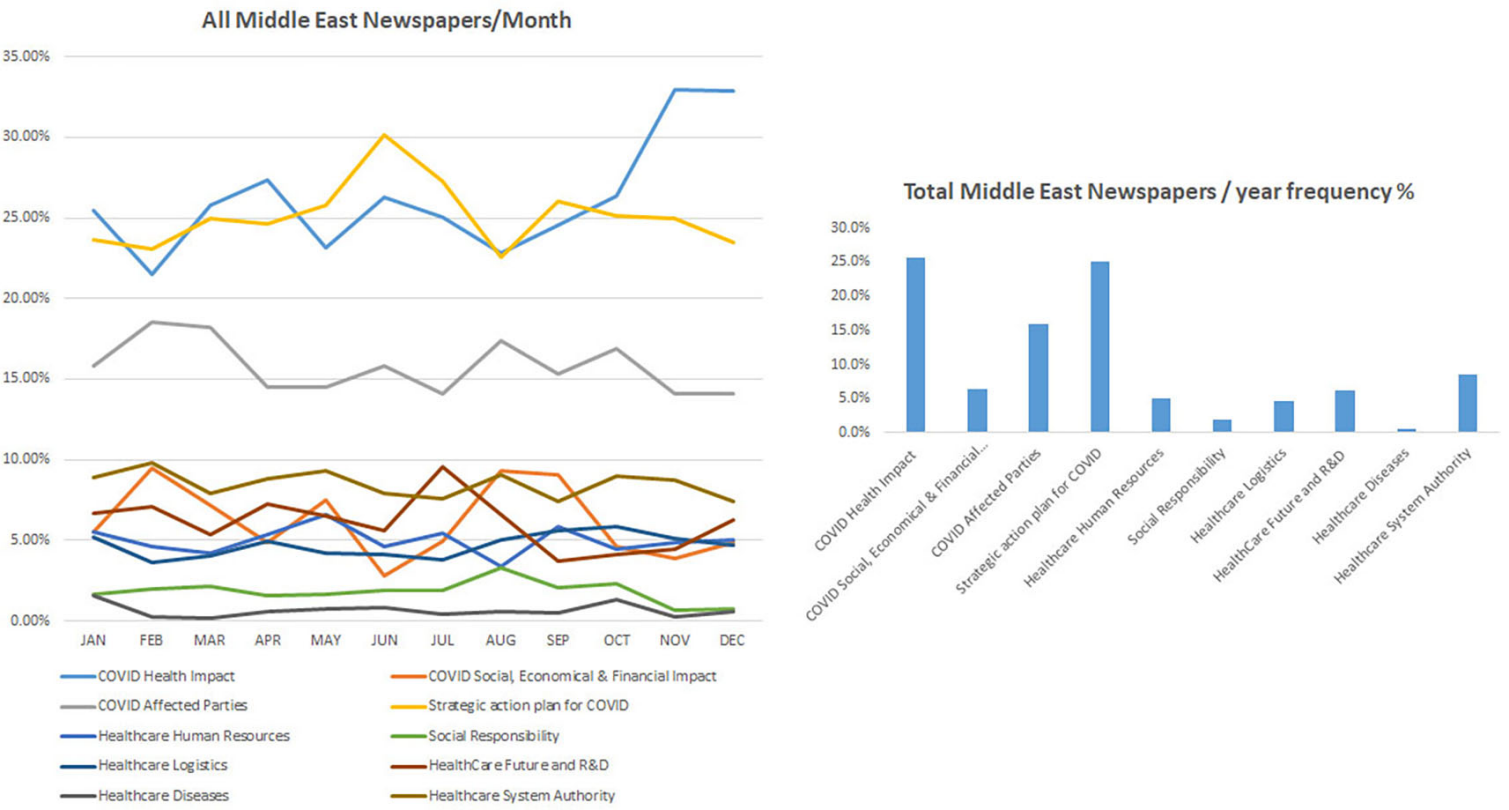
Trendline and frequency of categories of all Middle East newspapers per month and year respectively.

**Figure 3.**
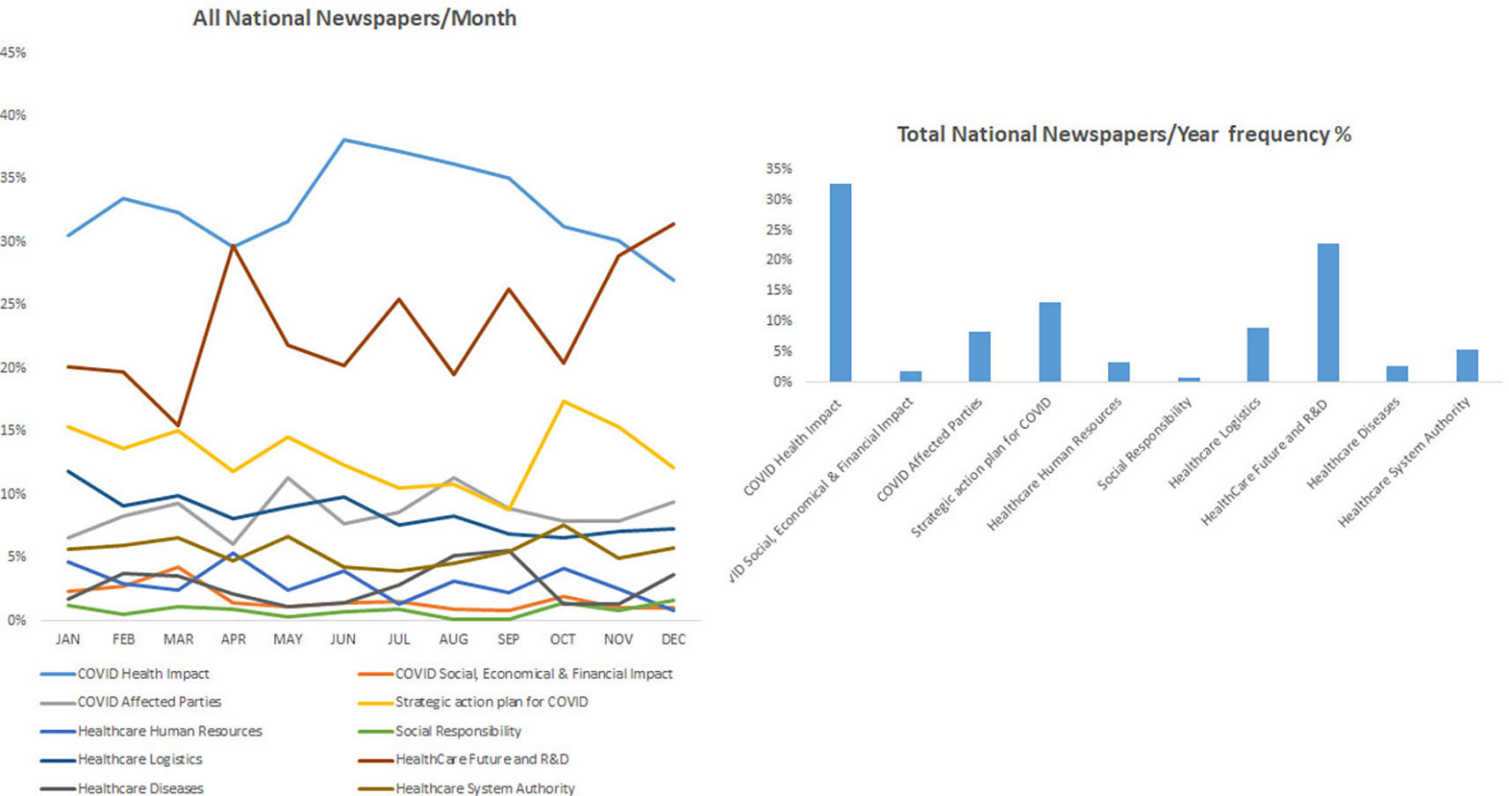
Trendline and frequency of categories of all national newspapers per month and year respectively.

The development of new global and local COVID-19 prevention strategies (curfews, use of masks, limiting social contact, use of sanitizers) enabled the healthcare system to effectively use its scarce resources to serve the largest number of infected patients, ^35^^,^^36^ thus preventing the collapse of the healthcare systems. COVID-19 fightback strategies became a popular and a frequently reported healthcare issue, thus shifting attention from other diseases and negatively influencing the communication outflow for chronic illnesses ( *e.g.* diabetes, cancer, kidney diseases). ^37^^–^^39^ The latter finding was highlighted with the reduced frequencies of “health diseases” that did not bypass 10% in international newspapers and was almost inexistent in Middle East and national periodicals ( Figures 1, 2 and 3).

The scarcity of resources worldwide subjected countries with strong healthcare systems to experience COVID-19 transfer waves originating from less developed countries. At first, the USA, China, and Europe were interested in fighting COVID-19 within their borders. Due to the massive movement of people, such a strategy was not effective. Therefore, it was of utmost importance to find better and sustainable solutions not only to stop COVID-19 waves but also to establish better health policies worldwide, thus limiting the spread of COVID-19. In addition, many countries worldwide were convinced that the spread of infectious diseases could not be limited by geographical boundaries thus collective international efforts must be initiated in order to face not only COVID-19 but also future challenges. ^40^

Initially, in the beginning of data collection, it was observed by the authors that COVID-19 influence on the healthcare was worldwide and was not country specific. All countries were significantly affected by COVID-19 with various degrees, therefore it was expected that COVID-19 related news on health care were at the forefront. Our data corroborated these findings and further showed that the keyword “healthcare” was the most frequent keyword repeated in the study. A case in point, strategies to fight the pandemic started to surface in the periodicals in response to the collapsing Italian healthcare system in 2020. Accordingly, the category “strategic action plan for COVID-19” was the mostly reported with frequencies bypassing 25% international newspapers, 30% in Middle East newspapers and 35% in Lebanese periodicals. Fear from future collapse of other healthcare systems fueled the news with the importance of taking measures to fight the pandemic without compromising the efficacy of the healthcare system. Accordingly, strategies to reduce disease spread through vaccination as an alternative tool to reduce the pressure on the healthcare system were among the earliest measures adopted by the healthcare authorities. ^41^ While strategies and policies to fight COVID-19 where among the repeated keywords in the study, other important keywords such as “healthcare budget” were not highly reported in the media and showed low frequency of reporting as compared to other keywords. All the chosen periodicals showed similar trend lines (health care, strategies), where the corresponding categories were reported at frequencies ranging 5–15% among all newspapers.

Discrepancies were found among surveyed periodicals, where the keyword “research and development” was frequently reported in international periodicals while it was mostly missing in local and regional periodicals ( Figures 1, 2 and 3). The latter findings reflect the trend of developed countries to use R&D in fighting diseases and providing policies and procedures to develop their healthcare interventions, which is lacking in other studied countries. Also, the keyword “vaccines” appeared as one of the most frequent keywords in the study and the earliest in international periodicals ( Figure 1). Accordingly, the keyword “vaccines” was not reported in regional and national periodicals at the beginning of the pandemic. The impact of COVID-19 on the healthcare system and the economies appeared early months of the studied year in the international periodicals where the keyword “healthcare” was reported at a very high frequency of 60% compared to the regional and local periodicals (around 30%). The above-mentioned data, showed that healthcare news are described first in international periodicals then appear in local and regional periodicals setting a trend of considering international periodicals as a source of data. Furthermore, theses data highlight the fact that local and regional periodicals are quick followers and stay current with the news as soon as it is published in the international periodicals. Accordingly. They use international periodicals as source of information, they identify the piece of information they are interested in and get it translated and immediately (1-2 days max) published in the local or regional media.

The limitation of the study is associated with the human factor as decoding the periodicals manually which may have created discrepancies among the coders. Such coding processes can be influenced by all the well-known human factors including emotions, language barriers, efficiency, and understanding of the objectives. However, reducing the workload on each team member and providing proper training before starting the research process greatly contributed to minimizing such bias. While the content analysis research approach is well-known in the literature, to our knowledge it is the first study that tackles COVID-19. Interestingly, in the era of artificial intelligence and the availability of powerful search engines on the web, the human factor remains essential in deciphering the quality and quantity of information needed to formulate proper policies that are compatible with nowadays challenges. Lastly, we believe that periodicals whether they are published at the local, regional or international level were found to have an important role in guiding and raising awareness about strategic health care issues that could be of great importance globally.

## Conclusions

Our data showed that international periodicals served as sources of COVID-19 data input for local and regional COVID-19 news. Our data concluded that international periodicals act as “trendsetters” whereas regional and local news were followers. This is expected given the scarcity of resources allocated to R&D COVID-19, in addition to the lack of policies and health task forces to face challenges such as COVID-19 pandemic. Therefore, global media can be a crucial source of up-to-date healthcare policies originating for low income countries in order to respond fast and actively to future local and global health care challenges.

## Data Availability

Zenodo: Content Analysis Data set. https://doi.org/10.5281/zenodo.7785825. ^42^ Data are available under the terms of the Creative Commons Attribution 4.0 International license (CC-BY 4.0).
