# Supplementary material for: Assessment of the global healthcare industry during COVID-19 pandemic: A content analysis approach
Source: F1000Res. 2024 Apr 24;12:1310. Originally published 2023 Oct 11. [Version 2] doi: 10.12688/f1000research.132486.2 (PMC11809683; doi:10.12688/f1000research.132486.2)
Supplement: Peer review history

# Reviewer report: Reviewer response for version 2

**DOI:** 10.5256/f1000research.164710.r344382

**Published:** 2024-12-14

**Authors:** Omolara Oluseun Juba, Idowu Oluwaseun David Juba

**Affiliations:** 1 Cardiff Metropolitan University, Cardiff, Wales, UK; 2 Finance, Ekiti State University (Ringgold ID: 92958), Ado Ekiti, Ekiti, Nigeria

**This article provides a valuable contribution to understanding healthcare media coverage during the COVID-19 pandemic**.

 B **elow are the highlighted few points that makes this article strong.**

**1. Timely Relevance**

 The study addresses a critical period of global healthcare upheaval, providing valuable insights into media coverage and its influence during the COVID-19 pandemic.

**2. Methodological Rigor**

 Detailed descriptions of data collection and coding ensure replicability and methodological transparency.

**3. Global Scope**

 The inclusion of periodicals from diverse regions provides a comparative view of media coverage and highlights disparities in healthcare reporting.

**4. Data Accessibility**

 The authors have made the dataset available under a Creative Commons license, ensuring transparency.

**5. Interdisciplinary Collaboration**

 The study is strengthened by its interdisciplinary team of EMBA and medical students.

Is the work clearly and accurately presented and does it cite the current literature?

Yes

If applicable, is the statistical analysis and its interpretation appropriate?

Yes

Are all the source data underlying the results available to ensure full reproducibility?

Yes

Is the study design appropriate and is the work technically sound?

Yes

Are the conclusions drawn adequately supported by the results?

Yes

Are sufficient details of methods and analysis provided to allow replication by others?

Yes

Reviewer Expertise:

Author of multiple research publications with citations on topics such as workplace health and safety programs, Strategies for dementia care. Impact of global healthcare policies during pandemics. Expertise in conducting and analyzing systematic reviews and content analysis.

We confirm that we have read this submission and believe that we have an appropriate level of expertise to confirm that it is of an acceptable scientific standard.

# Reviewer report: Reviewer response for version 2

**DOI:** 10.5256/f1000research.164710.r269278

**Published:** 2024-06-06

**Authors:** Clara Abou Samra

**Affiliations:** 1 American University of Beirut, Beirut, Lebanon

No new comments.

Is the work clearly and accurately presented and does it cite the current literature?

Partly

If applicable, is the statistical analysis and its interpretation appropriate?

Not applicable

Are all the source data underlying the results available to ensure full reproducibility?

Partly

Is the study design appropriate and is the work technically sound?

Yes

Are the conclusions drawn adequately supported by the results?

Partly

Are sufficient details of methods and analysis provided to allow replication by others?

Partly

Reviewer Expertise:

health policy

I confirm that I have read this submission and believe that I have an appropriate level of expertise to confirm that it is of an acceptable scientific standard.

# Reviewer report: Reviewer response for version 1

**DOI:** 10.5256/f1000research.145410.r224457

**Published:** 2024-02-13

**Authors:** Clara Abou Samra

**Affiliations:** 1 American University of Beirut, Beirut, Lebanon

The study aims to analyze the impact of the COVID-19 pandemic on the global healthcare industry using Content Analysis (CA) of periodicals from different regions. The researchers collected data throughout 2021, applying a conventional CA approach to identify emerging patterns and trends in healthcare-related content.

 The study is well presented. However, the literature review lacks depth and in some cases citations. It is suggested to incorporate a more comprehensive literature review with studies on content analysis in health research and the impact of COVID-19 on healthcare systems. While the methodology is well-detailed, more information on the training of coders, addressing potential biases and ensuring consistency in data collection is needed. Providing additional details in these areas would enhance the replicability of the study. It is also suggested to provide details on how others can access the online repository containing source data. This is essential for ensuring transparency and replicability.

 Although the conclusions align with the results, the study lacks an in-depth discussion of limitations, potential biases, and implications. The claim that international periodicals act as "trendsetters" needs more exploration and discussion.

Is the work clearly and accurately presented and does it cite the current literature?

Partly

If applicable, is the statistical analysis and its interpretation appropriate?

Not applicable

Are all the source data underlying the results available to ensure full reproducibility?

Partly

Is the study design appropriate and is the work technically sound?

Yes

Are the conclusions drawn adequately supported by the results?

Partly

Are sufficient details of methods and analysis provided to allow replication by others?

Partly

Reviewer Expertise:

health policy

I confirm that I have read this submission and believe that I have an appropriate level of expertise to confirm that it is of an acceptable scientific standard, however I have significant reservations, as outlined above.

# Response

**Published:** 2024-04-05

**Authors:** Wissam Faour

**Affiliations:** Pharmacology, Lebanese American University School of Medicine, Byblos, Lebanon

On behalf of the authors, I would like to thank you and the reviewers for their effort and constructive comments. Please find below our answers to each of the comments raised by the authors.

 The study aims to analyze the impact of the COVID-19 pandemic on the global healthcare industry using Content Analysis (CA) of periodicals from different regions. The researchers collected data throughout 2021, applying a conventional CA approach to identify emerging patterns and trends in healthcare-related content.

 The study is well presented. However, the literature review lacks depth and in some cases citations.

 We thank the reviewer for this comment. Regarding the introduction, it was meant to introduce the methodology and its importance to perform the search related to the topic. We tried to be as much specific as possible to reduce potential redundancy and unnecessary increase in length. In addition, we included 29 references out of 42 in the introduction to make it rich and focus

 It is suggested to incorporate a more comprehensive literature review with studies on content analysis in health research and the impact of COVID-19 on healthcare systems.

 We thank the reviewer for this comment, and we add the following references along with the corresponding paragraph in the introduction as requested:

 26.       Dolatkhah, R., et al., *Content analysis of identity challenges in patients with haemophilia: A qualitative study.* Nurs Open, 2021. **8**(3): p. 1444-1451.

 27.         Karanikola, M.N.K., *Content Analysis in Critical and Emergency Care: A Discussion Paper.* sgrwfccn, (1): p. 8-23.

 28.         Gyberg, A., et al., *Struggling for access to appropriate healthcare services: A qualitative content analysis of patient complaints.* Journal of Advanced Nursing, 2023. **79**(10): p. 3748-3759.

 29.         Reifegerste, D. and A. Wiedicke, *Content Analysis in the Research Field of Health Coverage*, in *Standardisierte Inhaltsanalyse in der Kommunikationswissenschaft – Standardized Content Analysis in Communication Research: Ein Handbuch - A Handbook*, F. Oehmer-Pedrazzi, et al., Editors. 2023, Springer Fachmedien Wiesbaden: Wiesbaden. p. 179-191.

 While the methodology is well-detailed, more information on the training of coders, addressing potential biases and ensuring consistency in data collection is needed. Providing additional details in these areas would enhance the replicability of the study

**We thank the reviewer for the comments, as such we added the following section to the methodology to further clarify the used method.**

**Training the coders and search procedure**

 Two weeks prior to the initiation of the coding process, coders went through several intensive training sessions. Over time and with ongoing practice, coders developed the technical skills of matching. The 13 coders were broken down into 3 clusters, cluster 1 (International periodicals) was composed of 5 coders, cluster 2 (ME) and cluster 3 (Lebanese), both clusters were composed of 4 coders each respectively. Coders visited the library where all periodicals for five years period was awaiting for them. Each coder was issued a specific periodical for one year.  The coders were first instructed to select all healthcare related articles, then look for keywords related to “healthcare”. Once the keyword was identified it will be counted as one data point. As example, any article in the periodical containing a healthcare related keyword “Vaccine” it is consider as one data point for the vaccine keyword.  Similarly keywords such as “Hospital” if it exists in the article it adds one point to the hospital keyword count. The presence of the word is counted once only. To improve data validity and reliability the number of times the keyword is repeated in the article was not counted. Data was collected from all periodicals once a week throughout the investigation period of 2021. Coders were trained, then assigned to investigate specific periodicals in each cluster. Regular meetings were held to monitor the coders’ compliance with the objectives of the study and the use of agreed upon coding schemes. Results represent data for each region of the world. Further, all regions were compressed together, and the global data set emerged. The result section of the compressed global data obtained after regrouping the keywords in sets resulted in 10 categories which represent the most frequently reported healthcare issues during 2021 and are defined in

 It is also suggested to provide details on how others can access the online repository containing source data. This is essential for ensuring transparency and replicability.

 The link to the repository is added at the end of the references and numbered 42

 Although the conclusions align with the results, the study lacks an in-depth discussion of limitations, potential biases, and implications. The claim that international periodicals act as "trendsetters" needs more exploration and discussion.

 Answer: We than the reviewer for this comment, and the following paragraph of the discussion section is modified to answer the reviewer’s comment:

 Discrepancies were found among surveyed periodicals, where the keyword “research and development” was frequently reported in international periodicals while it was mostly missing in local and regional periodicals ( Figures 1, 2 and 3). The latter findings reflect the trend of developed countries to use R&D in fighting diseases and providing policies and procedures to develop their healthcare interventions, which is lacking in other studied countries. Also, the keyword “vaccines” appeared as one of the most frequent keywords in the study and the earliest in international periodicals ( Figure 1). Accordingly, the keyword “vaccines” was not reported in regional and national periodicals at the beginning of the pandemic. The impact of COVID-19 on the healthcare system and the economies appeared early months of the studied year in the international periodicals where the keyword “healthcare” was reported at a very high frequency of 60% compared to the regional and local periodicals (around 30%). The above-mentioned data, showed that healthcare news are described first in international periodicals then appear in local and regional periodicals setting a trend of considering international periodicals as a source of data. Furthermore, theses data highlight the fact that local and regional periodicals are quick followers and stay current with the news as soon as it is published in the international periodicals. Accordingly. They use international periodicals as source of information, they identify the piece of information they are interested in and get it translated and immediately (1-2 days max) published in the local or regional media

  

- **Is the work clearly and accurately presented and does it cite the current literature?**

Partly

 Answer: more references were added to the introduction to make it rich

- **Is the study design appropriate and is the work technically sound?**

Yes

 Thanks for the comments

- **Are sufficient details of methods and analysis provided to allow replication by others?**

Partly

 Answer: we added an entire section describing the methodology as requested

- **If applicable, is the statistical analysis and its interpretation appropriate?**

Not applicable

- **Are all the source data underlying the results available to ensure full reproducibility?**

Partly

 Answer: The data are available in the repository referenced in the paper

- **Are the conclusions drawn adequately supported by the results?**

Partly

 Answer: We clarified the points that requires further discussion as requested by the reviewers
